# Cultural variation between neighbouring communities of chimpanzees at Gombe, Tanzania

**DOI:** 10.1038/s41598-019-44703-4

**Published:** 2019-06-04

**Authors:** Alejandra Pascual-Garrido

**Affiliations:** Primate Models for Behavioural Evolution Lab, Institute of Cognitive and Evolutionary Anthropology, School of Anthropology and Museum Ethnography, University of Oxford, 64 Banbury Road, Oxford OX2 6 PN, United Kingdom

**Keywords:** Biological anthropology, Animal behaviour

## Abstract

Comparative animal studies have revealed the existence of inter-group differences in socially learned behaviours – so-called cultural variations. However, most research has drawn on geographically and thus environmentally separated populations, rendering it difficult to exclude genetic or ecological influences. To circumvent this problem, the behaviour of neighbouring groups from the same population can be juxtaposed - an approach which in wild chimpanzees (*Pan troglodytes*) has revealed cultural differences in the use of nut-cracking and ant-dipping tools. Here I apply archaeological methods to extend this approach to compare the qualities of termite fishing tools used by wild chimpanzees by comparing the neighbouring Kasekela and Mitumba communities at Gombe Stream National Park, Tanzania. While no identifiable differences existed between the available plant species and associated vegetal components, members of the Kasekela community selected a larger array of raw materials and manufactured significantly longer and wider tools compared with the Mitumba community. Thus, cultural knowledge is reflected in differentiated behaviour on a small spatial scale. This study emphasizes the use of archaeological methods to identify cultural variation among living chimpanzee communities, adding to the growing research operating within the new field of Primate Archaeology.

## Introduction

The human ability to transmit knowledge socially leads to the development of local cultures, which shapes behavioural differences between populations and promotes group cohesion^1^. In the biological sciences, a cultural behaviour is one that is transmitted repeatedly through social or observational learning, to become a population-level characteristic^2^. Not all workers agree that nonhuman traditions can be appropriately termed culture (see McGrew^3^ for a review of the debate), but I will follow Whiten *et al*.^2^ inclusion of chimpanzee traditions as cultural behaviour (culture in this case defined as population-specific socially transmitted behaviours). By this definition, there is evidence of cultural variation in a vast array of animals including primates^2,4–6^, cetaceans^7^, birds^8^, and even fish^9^. The principal method used to establish culture in wild animal populations is the method of exclusion^2^. In this method, researchers identify geographically variable behavioural patterns across study sites and seek to identify those differences that cannot be attributed to ecological or genetic explanations, but instead are underpinned by social (not individual) learning^9^. However, this method can be problematic: how can one conclusively exclude the influence of environmental and genetic factors^10–13^, especially between distant populations? Furthermore, even when differences in behaviour patterns between groups can be explained by culture ‘alone’^3,6,14^, this does not mean that environment and genes have no influence on cultural processes^15^ - obviously, without genes and an environment to build an organism, culture could never develop at all.

In order to minimize the influence of these two factors on non-human material culture, one can focus on neighbouring communities that live in close proximity to one another. In chimpanzees, cultural differences between neighbouring communities have been proposed to include tool use behaviours, social customs and foraging styles^3,16–19^. By studying genetically similar groups living under very similar environmental conditions, fine scale cultural differences between groups can more easily be identified.

Within animal culture studies, variations in extractive foraging behaviours in chimpanzees have provided some of the best-accepted cases of socially-transmitted inter-populational differences^2,20^, suggesting that the observed behavioural variation in the wild is an expression of culture in chimpanzees^21,22^. A recent study of the variation in foraging behaviour between neighbouring chimpanzee communities took place in the Taï Forest, Cote d’Ivoire, where the chimpanzees hammer open *Coula edulis* nuts using percussive technology^23^. Community differences attributed to culture were found in hammer material choice and hammer size^18^. Furthermore, diversity in tool selection persisted over several decades due to immigrants abandoning their previous tool preferences and adopting the pattern of their new community^24,25^. A similar approach in the forests of Kalinzu, Uganda, revealed cultural differences between neighbouring chimpanzee communities in the length of tools used to dip for army ants (*Dorylus* spp.)^19^. This study, however, did not take into account the type of material used for tool manufacture. Given that the type of material employed may be influenced by social input^26–28^, and determine the final characteristics of the tool^29,30^, I here extend this approach to investigate differences in raw material choice and tools employed for termite-fishing between two neighbouring chimpanzee communities in East Africa.

Termite fishing is one of the most widespread chimpanzee tool use behaviours^20^. The techniques used vary regionally: some chimpanzee communities, such as the ones from the research site in this study use a simple probe inserted into an exit-hole of the mound^31^, while others use more than one type of tool in succession. In the Goualougo Triangle, Republic of Congo, this includes the use of stout sticks to access the insects’ underground chambers; these techniques may resemble the way early humans used bone and likely stick tools to access termite mounds^30,32,33^. Materials used to make tools likewise vary between populations: while the use of bark is seen in both west and east Africa, it appears to be absent from the few Central African populations that have been studied^34^ (but see Hicks *et al*.^35^ for evidence of ‘bark scoop’ for ponerine ants in the Bili-Uéré chimpanzees of North Democratic Republic of Congo). Chimpanzees obtain tool materials from different parts of plants, including bark, herb, grass, leaves, twigs, vines, petioles and sedges^3,36–39^. While some populations use a variety of species and plant parts as materials^36^, others use exclusively one, even when other suitable materials are more abundant, suggesting strong preferences in these populations^40,41^. While such preferences can in some cases be explained by the function of the tool (i.e. probe versus puncture)^30^, in other cases certain materials seem to be arbitrarily chosen and thus more likely to be attributed to social influences^40^.

Chimpanzees acquire their termite fishing skills primarily through observation of their mothers and maternal relatives during their early years of life^42^. Despite missing evidence of active teaching in termite fishing (but see Musgrave *et al*.^27^ for tool transfers as a form of teaching), some aspects of this behaviour, i.e. tool length and material selectivity, may be influenced by social learning^27,42–45^, and thus be considered candidates for group-specific behavioural variants^2^, while others seem to be influenced by environmental factors, such as the behaviour of the termite prey^45^. Variation in termite fishing tools between Goulaougo chimpanzees and chimpanzees living at La Belgique, Cameroon, were found in tool length and width. While some aspects of these variations were linked to the characteristics of the targeted termite prey, i.e. nest structure, other aspects, including material selectivity, were likely influenced by social factors^45^. Furthermore, adjacent communities of chimpanzees in the Goulaougo Triangle manufactured tools of different length^39^, however without detailed analysis of termite prey and raw material available, it was not possible to attribute these differences to social customs.

In this study, I investigated differences in the characteristics of termite fishing tools and raw material employed between neighbouring chimpanzee (*Pan troglodytes schweinfurthii*) communities living in similar habitats in Gombe Stream National Park, western Tanzania. The two study communities, Kasekela and Mitumba, habitually engage in different types of tool use associated with extractive foraging for insects, including termite fishing^31,46^. Chimpanzees at Gombe ‘fish’ for *Macrotermes* termites year-round but concentrate their efforts during the most productive season (Oct-Dec), which coincides with the annual reproductive and dispersal cycles of the termites, when the termites are more accessible^47^. Abandoned fishing tools are regularly recovered during these months, while they are scarce during the dry season^48^. Regular female migration occurs between the Mitumba and Kasekela communities, with the potential for the exchange of socially-transmitted techniques between communities^49^, thus providing an excellent opportunity to investigate cultural differences in termite fishing tools between adjacent communities.

In order to maximise the information obtained from records of past behaviour, and despite the fact that both study communities have been the subject of numerous detailed behavioural investigations, including in termite fishing^42–44,47,49^, in this study, I used exclusively traditional archaeological methods. Primate archaeology is a new field of research that investigates material cultures of non-human primates to improve interpretations of early archaeological sites, with respect to the fact that most implements were made from perishable plant material^50^. I recorded data on tool characteristics used by members of both neighbouring communities at targeted termite mounds. These tool characteristics included length, width, tool material and plant species used, as well as termite species targeted. In order to test for tool material selection, I conducted an availability study of raw material near the focal termite mounds^15,40^. I examined the relationship between tool characteristics and various plants used as tool material.

## Results

### Termite mounds

All targeted mounds were from *Macrotermes bellicosus* except for two *M*. *bellicosus* mounds at Kasekela which were also occupied by two additional *Macrotermes* residents: *M*. *michaelseni* and *M*. *subhyalinus* (Table 1). The mean number of tools recovered per visit at each of the termite mounds was 5.0 (SD = 4.6, n = 74, range: 1–24) for Kasekela and 6.2 (SD = 4.5, n = 43, range: 1–19) for Mitumba. The full null model comparison revealed no differences between the two communities in the number of tools recovered per visit (X² = 1.8855, df = 1, *P* = 0.1697). The model indicates that the larger mounds were associated with more tools per visit (estimate = 0.19, X² = 3.92, df = 1, *P* = 0.048).

**Table 1:** Recovered tools at study mounds targeted for termite fishing by the two neighbouring chimpanzee communities at Gombe Stream National Park, Tanzania (termite mound, targeted termite species, tool material, tool species).

Community	Termite mound	Termite Species	Tools (n)	Tool Material				Tool Species	
				Bark (n)	Twig (n)	Vine (n)	Grass (n)	*M*. *poggei* (n)	*D*. *lucida* (n)
Kasekela	GTM008	*M. bellicosus & M. michaelseni*	88	56	29	2	1	23	1
	GTM009	*M. bellicosus*	62	48	13	1		13	1
	GTM010	*M. bellicosus*	24	9	15			5	5
	GTM011	*M. bellicosus, M. michaelseni & M. subhyalinus*	92	43	49			39	40
	GTM012	*M. bellicosus*	81	18	39	22	2	8	21
	GTM013	*M. bellicosus*	41	20	20		1	5	3
	GTM014	*M. bellicosus*	23	6	16		1	11	7
	Subtotal		411	200	181	25	5	104	78
	% tool material			48.7	44.0	6.1	1.2		
Mitumba	MIT003	*M. bellicosus*	53	8	45			14	39
	MIT005	*M. bellicosus*	11	2	9			2	9
	MIT006	*M. bellicosus*	61	8	53			10	42
	MIT007	*M. bellicosus*	109	14	95			24	75
	MIT011	*M. bellicosus*	29	9	20			11	18
	MIT012	*M. bellicosus*	5	4	1			61	183
	Subtotal		268	45	223	0	0		
	% tool material			16.8	83.2	0	0		
	Total		679	245	404	25	5		

### Tool characteristics

**Figure 1:**
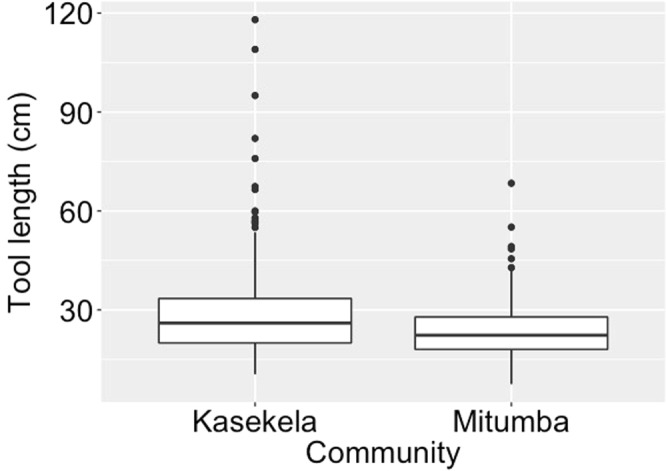
Termite fishing tool length (cm) in the two neighbouring communities at Gombe. Boxplots indicate upper and lower quartile, with median as thicker horizontal line. Whiskers indicate maximum and minimum data range (excluding outliers). Circles show individual outliers. *X² = 6.38, df = 1, *P* = 0.012.

Kasekela tools were significantly longer 28.1 cm (SD = 12.8, n = 405, range: 10.5–118 cm) and wider 1.9 mm (SD = 1.1, n = 404, range: 0.1–7.6) than Mitumba tools with length 23.5 cm (SD = 8.2 cm, n = 267, range: 7.5–68.4) and width 1.4 mm (SD = 0.6, n = 267, range: 0.6–5.7). The full null model comparison revealed that the two communities differed significantly in tool length produced, when controlling for tool material and species (X² = 6.38, df = 1, *P* = 0.012). Specifically, Kasekela community produced longer tools than Mitumba community (estimate_Mitumba_ = −4.66, Fig. 1 & Supplementary Fig. S1). Tool material did not influence tool length, but tools fashioned from *G*. *forbesii* were longer than from the other two species (estimate_G.forbesii_ = 10.89, X² = 25.60, df = 1, *P* < 0.001, Supplementary Tables S1, S2).

**Figure 2:**
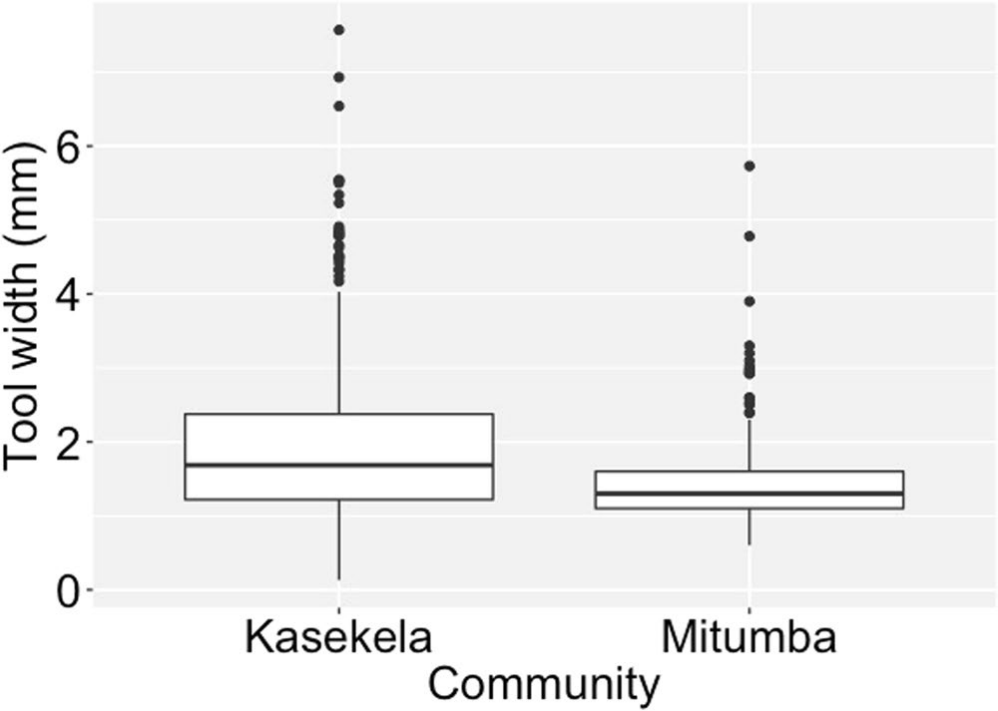
Termite fishing tool width (mm) in the two neighbouring communities at Gombe. Boxplots indicate upper and lower quartile with median as thicker horizontal line. Whiskers indicate maximum and minimum data range (excluding outliers). Circles show individual outliers. *X² = 4.42, df = 1, *P* = 0.036.

As with the length, the full null model comparison showed that the two communities differed significantly in tool width (X² = 4.42, df = 1, *P* = 0.036), with Kasekela producing wider tools than Mitumba (estimate_Mitumba_ = −0.20, Fig. 2 & Supplementary Fig. S1). Tools made from *G*. *forbesii* were wider than those made from *M*. *poggei* (estimate_G.forbesii_ = 1.37, X² = 84.96, df = 2, *P* < 0.001) which were in turn wider than *D*. *lucida* (estimate_D.lucida_ = −0.23, Supplementary Table S1). Bark tools were significantly wider than twig tools (estimate_twig_ = −0.58, X² = 29.08, df = 1, *P* < 0.001, Supplementary Table S2).

Comparing the two tool characteristics revealed that the length and width of tools were weakly, but significantly correlated (r = 0.25, t = 5.64, df = 469, *P* < 0.001), so that wider tools were also generally longer.

### Tool material

**Figure 3:**
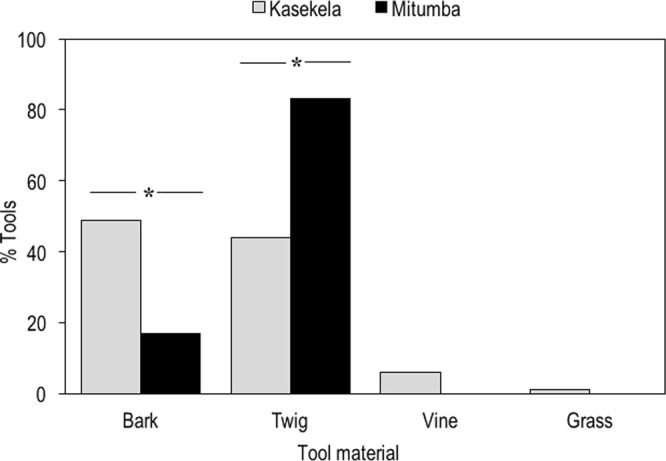
Proportion of tool material used by the two neighbouring communities at Gombe. Type of raw material used (bark, twig, vine, grass) for Kasekela community (grey) and Mitumba community (black). *X^2^ = 107.06, df = 3, *P* < 0.001.

Kasekela chimpanzees manufactured tools using different materials, with nearly half made from bark (48.7%) and nearly half made from twig (44.0%), and with a much smaller proportion made of vine (6.0%) and grass (1.2%) (Chi-square test with null-probability = 1/4: X^2^ = 303.46, df = 3, *P* < 0.001)^41^ (Table 1 & Fig. 3). In contrast, Mitumba chimpanzees manufactured their tools mostly from twigs (83.2%) and with a much lower proportion from bark (16.8%) (X^2^ = 504.45, df = 3, *P* < 0.001). None of their tools were made from grass or vines (Table 1 & Fig. 3). The relative proportion of tool material used (bark, twig, vine and grass) differed significantly between the two communities (X^2^ = 107.06, df = 3, *P* < 0.001).

### Tool species

**Figure 4:**
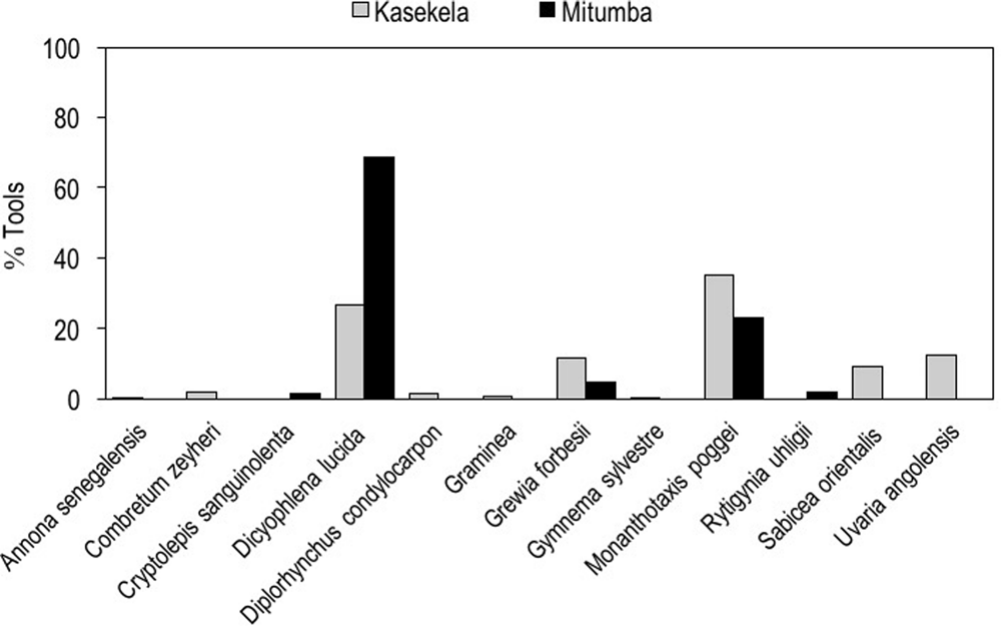
Proportion of plant species sourced for raw material by the two neighbouring communities at Gombe. Plant species sourced for Kasekela community (grey) and Mitumba community (black).

Tools used by the Kasekela community were made using 10 species of plants (see Table 2 from^41^), with significant differences between the proportion of species used (X^2^ = 395.15, df = 9, *P* < 0.001, n = 299). The largest number of tools was made from *M*. *poggei* (35.1%), followed by *D*. *lucida* (26.4%), *U*. *angolensis* (12.4%) and *G*. *forbesii * (11.7%) (Fig. 4).

Tools made by the Mitumba community were fashioned using five species, with differences between the proportion of species used (X^2^ = 437.38, df = 4, *P* < 0.001, n = 266). Their tools included species also sourced for the same material, bark and twigs, by the Kasekela community (*Dicyophlena lucida*, *Grewia forbesii* and *Monanthotaxis poggei*), as well as two species exclusive to this community: *Cryptolepis sanguinolenta* and *Rytigynia uhligii*. As was the case with Kasekela, Mitumba members made the largest proportion of tools from *D*. *lucida* (68.8%) and *M*. *poggei* (23.0%), although the frequency of use was reversed for these two species (Fig. 4), while *G*.* forbesii* was used in much lower proportion (5.0%).

### Availability of raw materials

I calculated the mean abundance of suitable raw material around mounds. At both sites, all plants counted per northwest 90° quadrant within 5m of targeted mounds provided at least one type of tool material, but there were differences among the type of raw material available (Kasekela: X^2^ = 561.25, df = 3, *P* < 0.001; Mitumba: X^2^ = 135.31, df = 3, *P < *0.001). For both communities, twigs were the most abundant material near mounds (Kasekela: 87.1%; Mitumba: 71.8%), followed by bark (Kasekela: 48.6%; Mitumba: 54.8%), and grass (Kasekela: 11.6%; Mitumba: 28.2%), while vines were present only at Kasekela (1.3%) (Table 2).

**Figure 5:**
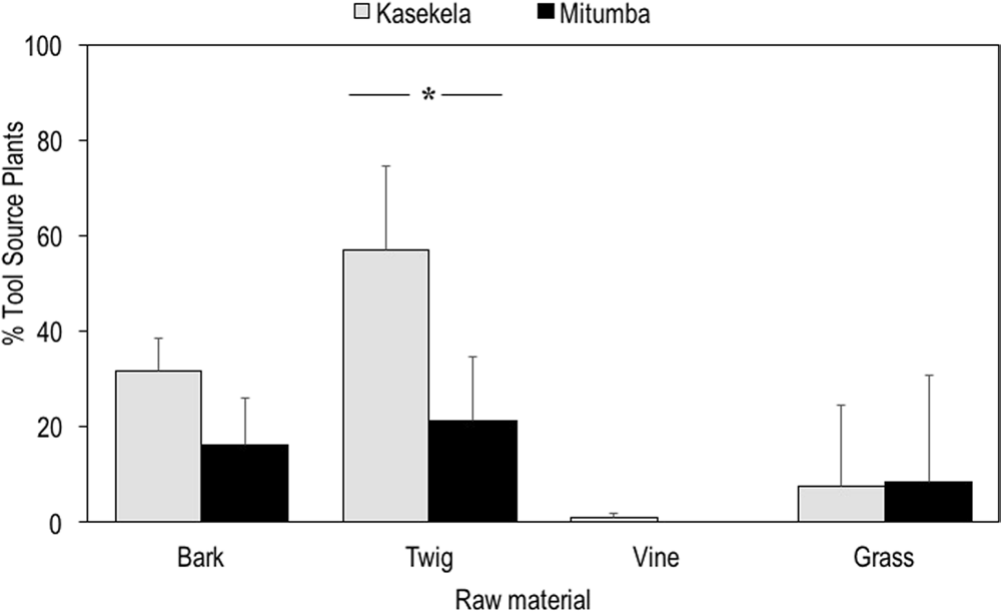
Mean abundance of suitable raw material sources near studied mounds in the two neighbouring communities at Gombe. Suitable raw material (bark, twig, vine, grass) for Kasekela (grey) and Mitumba (black).

Secondly, I compared the mean abundance of suitable bark, twig and grass source-species between Kasekela and Mitumba. The mean abundance of plants that could provide suitable bark per northwest 90°quadrant within 5 m of targeted mounds at Kasekela was 31.7 plants versus 16.2 at Mitumba, with no significant differences between the two communities (Independent Samples T-test: t = 3.5075, df = 9.8, *P* = 0.0057, Fig. 5). The mean abundance of twig sources at Kasekela was 56.9 plants versus 21.2 at Mitumba, with significant differences between the two sites (Independent Samples T-test: t = 4.0615, df = 10.2, *P* = 0.002153, Fig. 5). Mean abundance of grass was similar between communities, with 7.6 at Kasekela versus 8.3 at Mitumba (w = 22.5, *P* = 0.8468, Fig. 5).

**Table 2:** Main parameters of vegetation cover within a quarter section of a 5m radius circle of study mounds targeted by chimpanzees for termite fishing in the two neighbouring communities at Gombe (abundance of plants suitable as raw material for termite fishing probes and identified individual tool source species).

Site	Termite mound number	Total plants within quadrant (n)	Suitable plants to extract raw material (n)*	Suitable bark sources (n)	Suitable twig sources (n)	Suitable vine sources (n)	Suitable grass sources (n)	Plants of known sourced species (n)	% tool plants / suitable plants
Kasekela	GTM008	44	44	27	43	1	0	38	86.4
	GTM009	33	33	23	32	1	0	26	78.8
	GTM010	59	59	36	51	0	8	49	83.1
	GTM011	61	61	33	61	0	0	58	95.1
	GTM012	59	59	34	56	3	0	54	91.5
	GTM013	62	62	25	62	0	0	58	93.5
	GTM014	139	139	44	93	1	45	107	77.0
	Sum	457	457	222	398	6	53	390	
	Mean	65.3	65.3	31.7	56.9	0.9	7.6	55.7	86.5
	% relative to total plants		100						
	% relative to plants suitable as raw material sources			48.6	87.1	1.3	11.6	85.3	
Mitumba	MIT003	17	17	16	17	0	0	13	76.5
	MIT005	24	24	20	24	0	0	20	83.3
	MIT006	42	42	25	42	0	0	18	42.9
	MIT007	14	14	13	14	0	0	13	92.9
	MIT011	24	24	22	24	0	0	22	91.7
	MIT012	56	56	1	6	0	50	1	1.8
	Sum	177	177	97	127	0	50	87	
	Mean	29.5	29.5	16.2	21.2	0.0	8.3	14.5	64.8
	% relative to total plants		100						
	% relative to plants suitable as raw material sources			54.8	71.8	0.0	28.2	49.2	

*Please note that the same plant may provide more than one type of raw material.

Thirdly, at both sites, there were significant differences between the proportion of individual source species near targeted mounds (Kasekela: X^2^ = 1628.6, df = 14, *P* < 0.001; Mitumba: X^2^ = 131.44.6, df = 8, *P* < 0.001), with the two most abundant species being *M*. *poggei* (Kasekela: 49.5%, Mitumba: 23.0%) and *D*. *lucida* (Kasekela: 25.0%; Mitumba: 19.1%) – also the two species most represented in tool assemblages. Rare species near mounds included *G*. *forbesii* (Kasekela: 0.9%; Mitumba: 1.1%) and *U*. *angolensis* (Kasekela: 1.0%).

## Discussion

The characteristics of termite fishing tools and the type of tool material employed to prey on *Macrotermes* mounds differed between neighbouring chimpanzee communities in the Gombe Stream National Park. Kasekela members manufactured longer and wider tools than Mitumba individuals and used a larger array of materials to manufacture them. This raises the question as to why neighbouring communities living in such similar habitats differ in how they manufacture tools and the plant material they use to make them?

One possible explanation for the differential choice of tool material between the study communities is that the raw materials might not be equally available in the environment, and chimpanzees would be obliged to choose their plant materials according to local abundance. This is, at least for tool material, unlikely: First, despite raw material near mounds being, on average, more abundant for the Kasekela community (cf., Table 2), twigs were the most frequently-available material class for both communities. If the chimpanzees were choosing material only based upon abundance, we would expect chimpanzees from both communities to favour twigs over any other material. However, only Mitumba chimpanzees manufactured more of their tools from twigs, while Kasekela members used twigs and bark in almost equal proportion. Secondly, when analysing the tool species used, both communities made the majority of their tools from abundant species, such as *M*. *poggei* and *D*. *lucida*, but also from rare specimens such as *G*. *forbesii* and *U*. *angolensis*. Interestingly, these two species are also favoured for termite fishing by other populations of chimpanzees living not far away from Gombe in Tanzania: at Mahale and the Issa valley^40,41,51^. *Grewia* spp. is also sourced for termite fishing tools by chimpanzees at Mt. Assirik^52^ and Fongoli in Senegal^29^. That the same plant species are favoured by different communities of chimpanzees across Africa to manufacture termite fishing tools – including populations separated by more than 5,000 km – could be the result of their relative abundance in the landscape or because of their physical characteristics which make them ideal for fishing termites. Further studies to analyse the mechanical properties of tool species as well as detailed comparative data from other field sites are required in order to address this question.

Preferential usage of particular tool material and plant species for termite fishing has been reported for other termite fishing populations^30,34,36,38,52–56^. As pointed out by Teleki^48^ decades ago: “an intermediate range of (material) qualities must be selected if probing is to yield many termites”. While in some cases the most commonly used tool material is also the one that is most abundant near the termite mounds under study - and thus its use may be random and not preferential^52^ - in other cases the apes appear to be actively selecting preferred materials, regardless of ecological availability. For example, chimpanzees in the Goulaougo Triangle manufacture tools from herbs or trees not readily available near mounds^30^, while chimpanzees in the Issa valley exclusively use bark to manufacture their tools despite twigs being the most abundant material nearby (94%)^40^. At Gombe, where twigs were the most abundant material near mounds, only the Mitumba community manufactured the majority of their tools from this material - thus its use may be driven by ecology rather than a preferred material - while Kasekela chimpanzees used twigs and barks in almost equal proportions. That neighbouring communities living in similar environments favoured certain materials to make tools to prey on *M*. *bellicosus* despite similar availability of raw material, suggests a cultural preference for particular tool materials employed for termite fishing between neighbouring chimpanzee communities. Similar findings of preferences for particular tool materials, in this case for hammers used for cracking *Coula* nuts, were reported for chimpanzees in the Taï National Park^18^.

Tool length in Kasekela community (28.1 cm) is similar to that reported decades ago for the same community (30.7 cm)^36^, and, even if within the lower range of the spectrum, it resembles tool-lengths recorded at other sites in Tanzania (Table 3): Mahale B group (37.7 cm, 38.5 cm)^37,51^ and Issa (40.4 cm)^34^. However, tools made by the Mitumba community were significantly shorter than those manufactured by the Kasekela community, and well below the mean tool-lengths found at other sites in Tanzania (38.1 cm). Mitumba tool-length was the shortest (23.5 cm) reported for termite fishing tools across the chimpanzee range (Table 3). Likewise, Mitumba tools were significantly thinner (1.4 mm) than Kasekela tools (1.9 mm), and well below the mean tool width reported across other sites in Tanzania and the species’ range (Table 3).

**Table 3:** Termite fishing tool length, width and tool material across chimpanzee study sites. Mean termite fishing tool length in cm and tool width in mm, number of tools recovered, and prevalent type of tool material used across chimpanzee study sites.

Study site and sub-species	Length (cm)	SD	Number of tools	Most frequently used tool material	Width (mm)	SD	Number of tools	Most frequently used tool material	Proportion of material most frequently used (%)	References
*Pan troglodytes schweinfurthii*										
Gombe Mitumba, Tanzania	23.5	8.2	267	Twig	1.4	0.6	267	Twig	83	This study
Gombe Kasekela, Tanzania	28.1	12.8	405	Bark	1.9	1.1	404	Bark	48	This study
Gombe Kasekela, Tanzania	30.7	—	145	Grass	4.0	—	32	Grass	48	^36^
Mahale B group, Tanzania	54.6	—	97	Bark	—	—	—	—	—	^79^
Mahale B group, Tanzania	37.7	14.7	290	Bark	4.6	2.0	284	Bark	75	^37^
Mahale B group, Tanzania	38.5	10.3	25	Bark	3.1	1.0	25	Bark	100	^51^
Mahale K group, Tanzania	51.5	—	16	Bark	—	—	—	—	81	^79^
Issa, Tanzania	40.4	23.0	36	Bark	—	—	—	—	100	^34^
Mean	38.1				3.0					
*Pan troglodytes troglodytes*										
Goualougo, Republic of Congo	43.1	12.9	852	Herb	4.5	1.3	848	Herb	100	^39^
Guga, Republic of Congo	50.8	9.5	42	Herb	—	—	—	Herb	100	^55^
Lossi, Republic of Congo	54.3	11.6	107	Herb	3.9	0.9	78	Herb	100	^56^
Bai Hokou, Central African Republic	50.5	17.5	62	Twig/herbs	4.1	1.1	41	Twig/herb	100	^38^
Campo, Cameroon	30.5	—	4	Twig/stick	—	—	—	Twig/stick	100	^54^
Campo, Cameroon	44.4	5.0	16	Twig/stick	4.0	—	16	Twig/stick	—	^80^
La Belgique, Cameroon	54.7	11.4	129	Twigs	4.6	1.4	112	Twigs	100	^45^
La Belgique, Cameroon	49.4	10.5	150	Twigs	—	—	—	Twigs	100	^45^
Okorobiko, Republic of Equatorial Guinea	49.7	—	46	Twig/stick	11	—	46	Twig/stick	100	^36^
Belinga, Gabon	37.8	—	23	Twig/stick	3.5	—	23	Twig/stick	96	^53^
Mean	46.5				5.0					
*Pan troglodytes verus*										
Assirik, Senegal	32.5	—	173	Twig/stick	2.5	—	12	Twig/stick	47	^36^
Fongoli, Senegal, 2002	30	—	58	Grass	—	—	—	—	59	^29^
Fongoli, Senegal, 2003	34	—	75	—	—	—	—	—	—	^29^
Mean	32.2				2.5					

Variation in tool characteristics may be due to variation in the ecological availability of materials or possibly of the behaviour of the insect prey, whereas other aspects may be attributable to social influences and thus can be considered cultural^45,57^. Differences in the physical characteristics of termite fishing tools have been reported for chimpanzees living in the Congo Basin. In this region, tool types (puncturing sticks versus fishing probes) clearly reflect the specific nature of the structure of the termite mounds attacked^30,38,45,56,58^. Perforating a termite nest requires concentrated physical strength, and only a certain type of material is suitable for this task, while the more delicate task of probing which follows the initial brute-force puncturing favours the use of herbaceous stalks with an added brush-tip^30^. Intentional modifications to increase tool efficiency, such as the modification of Goualougo or Bai Hokou (Central African Republic) tools into brush tips to increase termite yields, may also be responsible for tool variation^30,38^. Another possible influence on the tool design may be the physical characteristics of the termite mound, such as size and surface thickness, or the behaviour of the insect prey^45^. Termites species with long mandibles grasp the tool with such a force that their mandibles cross and become locked, making it almost impossible for the chimpanzee to ‘sweep’ these termites from the fishing probe, being instead mouthed from the tool – a technique associated to longer tools^45^. Other aspects of tool characteristics seem to reflect social influences, including tool length and material choice^26,40,43,45^.

Within the scope of possible explanations for tool variation discussed above, which best explains the fact that the Mitumba chimpanzees use shorter and thinner tools than Kasekela? One explanation may be the influence of the materials used for tool manufacture^30^. Table 3 compares termite fishing tools reported from Gombe with those found in other sites with a focus on three variables: length, width and tool material. On average, termite fishing probes in the central subspecies of chimpanzees (*Pan troglodytes troglodytes)*, which exclusively use twigs and herbs, are longer and wider than those manufactured by chimpanzee communities living in east Africa, where bark is the material most commonly used. Based on this, we would expect Mitumba tools, mostly twigs, to be longer and wider than Kasekela tools – however the opposite is true. Still, conclusions from geographically distant populations are problematic since they cannot rule out the influence of genetics and/ or ecology^10,19^. Comparing tool characteristics across material types within communities show mixed results: while no significant difference in the lengths of tools recovered at Mahale (Bilenge) were found between bark and twig tools, tools made from bark were wider^37^. At Fongoli^29^, tools made from woody materials (vine, bark, twigs) were significantly longer (38.5 cm) than those made from grass (18.5 cm). Likewise, lengths of tools made from different materials by Goualougo chimpanzees (puncturing sticks, fishing probes and perforating twigs) differed in their length and width^39^. In the current study, the material type did not influence the tool length, but bark tools were significantly thicker than tools made from twigs, resembling the findings recorded at Mahale (Bilenge)^37^. That the two study communities differed significantly in their tool characteristics when controlling for material type, i.e. Kasekela bark tools were longer and wider than Mitumba bark tools, supports the presence of cultural differences on tool length and width between the communities, because the raw material used was identical.

The plant species used may also influence the tool’s characteristics^52,56^. At Goulaougo, this is clearly demonstrated by the apes manufacturing termite mound puncturing sticks mostly using *Thomandersia hensii*, a very straight and rigid tree species, while termite fishing probes were made mostly from *Sarcophyrnium* spp., a plant that has both long and flexible stalks^30^. The same holds true for chimpanzees at Bai Hokou: apes use plant species that offer the necessary physical characteristics for the manufacture of the different tool types. They choose pliable and slender material for termite fishing probes and straight and rigid sticks for perforating mounds^38^. Thus, it is not surprising that at Mitumba and Kasekela, species used for tool manufacture overlap. These species may hold as-of-yet-undetermined physical characteristics that make them ideal for termite fishing tools, such as flexible and resilient material capable of bending^36,48^. As tools recovered from Bai Hokou^38^ and Goualougo^30^, at Gombe certain tool species hold certain physical characteristics, i.e., tools made from *Grewia* spp. were significantly longer and wider (cf., Supplementary Table S1). However, the fact that differences between study communities were maintained when controlling for tool species, i.e. tools made from *M*. *poggei* spp. at Kasekela were longer and wider than tools made from the same plant species at Mitumba (cf., Supplementary Table S1), further suggests the likelihood of social influences on the tool design, because the tool species used was identical. Still, even in apparently similar habitats, it is almost impossible to entirely rule out micro-ecological features which might still be shaping some of the tool variation seen between the study communities. For example, plant species may grow wider shoots at Kasekela. If so, simply grabbing the nearest twig would still lead to a consistent difference between the communities. Future research is needed to examine in detail the physical characteristics of source species and investigate whether or not their characteristics, i.e. length of twigs, differ between the study communities.

Termite prey was identical for both communities - all targeted mounds were occupied by *M*. *bellicosus*, thus the choice of prey could not explain the differences in tool characteristics. Two mounds were also occupied by additional residents at Kasekela: *M*. *michaelseni* and *M*. *subhyalinus* (cf., Table 1) - all *Macrotermes* species targeted with the use of tools by chimpanzees elsewhere^36,37,51^. Occupancy of mounds by multiple residents, though a rare event, has been reported in the past^59^ and requires further investigation.

The physical structure of termite mounds and targeted species may also account for the variation seen between Kasekela and Mitumba tools^45^. However, the fact that differences between communities were maintained when controlling for mound size, rules out this possibility. The question of whether other aspects of structural variation between Kasekela and Mitumba mounds might be linked to the longer tools used by Kasekela chimpanzees, i.e. mounds at Kasekela might be thicker and chimpanzees manufacture longer tools to access them^45^, still remains to be tested.

These results provide persuasive evidence that cultural differences between neighbouring chimpanzees communities, as recorded for ant-dipping tools and hammers, extends to choices of plant material as well as the physical characteristics of termite fishing tools^18,19^. The question remains, however, as to how the local differences between neighbouring chimpanzee communities emerge and persist? Wild chimpanzees generally display male philopatry and female transfer, although juveniles of both sexes sometimes transfer with their mothers, and females, particularly at Gombe, sometimes remain in their natal community^47^. Because encounters between chimpanzee communities involving extra-group males are aggressive, and often fatal^47^, any cultural transmission between wild chimpanzee communities most likely occurs via female transfers (immigration). At Gombe, an immigrant female is thought to have been responsible for spreading tool-aided insectivory between Mitumba and Kasekela communities^49^. At Taï, a female chimpanzee immigrant was observed to adopt the hammer-selection preference of her new community^24^. Immigrating female chimpanzees have been reported to adopt the nut-cracking behavioural strategy specific to their new community within weeks, even when individual foraging success is compromised^60^. Such conformism in immigrant females can lead to long-lasting differences between communities, which may be maintained for at least 25 years, if not more^18,24,61^. Adopting the behavioural patterns of the ‘new’ community may provide physical and social benefits and a more rapid integration into the new group^24,62^. The drive to conform to the behaviour of the majority may result from immigrants seeking social information from knowledgeable group members about an unfamiliar environment, adopting potentially adaptive locally strategies^25,26^. As the prominent chimpanzee field researcher William McGrew clearly once stated: “*Imagine an extreme example*, *a Gombe female transferred to Goualougo*. *If she persisted in termite fishing Gombe-style*, *she would have no luck*, *as she would lack the other components*, *such as the penetrating tool*. *So*, *she would be smart to adapt to the local style as soon as possible*”. Conformity to local behavioural norms, that is, the tendency to adopt behavioural options that are common in the local population despite familiarity with, or the presence of alternative options, has also been documented in captive apes^63^ and monkeys^64^, as well as in the wild^65,66^. In humans, conformity is thought to have played a leading role in the evolution of culture, leading to the development of social systems characterized by stable in-group uniformity and between group diversity^67,68^. In M-group at Kalinzu, tool length for ant-dipping tools appears to have remained stable over time^19^. In the B-group of the Mahale Mountains, tool length for termite fishing tools reported for 1982–1983 (37.7 cm)^37^ closely resembles the tool length reported almost 35 years later (38.5 cm)^51^. The same seems to hold true for the Kasekela community at Gombe, where the tool length reported for termite fishing tools (30.7 cm) for 1972–1973^36^ closely resembles the tool lengths reported in this study (28.1 cm). Long-term data from both study communities are required to establish whether or not the observed difference in tool characteristics is maintained over time.

Termite fishing is a complex tool assisted foraging behaviour acquired by chimpanzees during their early years, with mothers being the primary model for learning^42–44^. Various forms of social input likely influence the acquisition of critical elements of this skill, i.e. choice of materials for construction, tools characteristics, and techniques of use, with stimulus enhancement or active facilitation (such as tool transfers) possibly playing a vital role^27,42–44^. At Gombe, female offspring preferred to insert tools of similar length to the ones their mother’s used and employed a technique similar to hers^43^. At Goulaougo, tool-using activity increased after tool transfers between mothers and offspring^27^. Given that some components of termite gathering may extend into the juvenile period and sub-adulthood^69^, the next step is to conduct longitudinal studies to examine social influences on the maintenance of these complex tool traditions in wild chimpanzees, including individual preferences in tool material choice and tool design, as well as possible changes of these in migrating females.

My findings at Gombe of differences in what are likely socially-transmitted behaviours between neighbouring communities provide further evidence that ‘subcultures’ of chimpanzees can be found between adjacent groups^18,19^ or even within communities^26,28^. In-group uniformity and between-group diversity reflects culture in humans^67,68,70^.Group cohesion and the desire to fit in has been suggested to be stronger in humans than in apes^71,72^, but it may also be important in wild-ranging chimpanzees^24,60^. More studies documenting divergent behavioural responses to similar ecological settings, including in choice of tool material or tool design, are required to fully understand the mechanisms responsible for and the ultimate functions of chimpanzee ‘subcultures’, which will aid in modelling the evolution of technology in early hominins^73^. This study adds to the growing research operating within the field of Primate Archaeology^50^ to identify records of cultural variation among neighbouring chimpanzee communities^18,25^ and highlights the importance of protecting the remaining habitats where chimpanzees live to ensure the continued survival of both the chimpanzee and their unique and diverse cultures^74^.

## Methods

### Study subjects and site

I carried out this research project in western Tanzania at Gombe Stream National Park (S4.67, E29.65), which is a site where multiple communities of chimpanzees fish for *Macrotermes* termites^47^. Gombe is a 35-km^2^ park on the eastern shore of Lake Tanganyika. Deep valleys fall from the rift escarpment to the lake. The valley bottoms are dominated by evergreen forests, woodland-covered slopes, and ridges carpeted with grasslands^47^. The Kasekela community (n = 55 in December 2017) resides in the centre of the Park and has been studied since 1960, with the majority of the individuals well habituated since 1966. The study of the northern community, Mitumba (n = 29 in December 2017) began in 1985, with most of the individuals habituated and individually recognised by 1994^49^.

### Data collection

In order to maximise the information contained from previously used tool use sites (termite mounds), data collection relied entirely on archaeological (indirect) methods, even if termite fishing has been the subject of numerous investigations during the last decades^42–44,47^. At each targeted mound, I collected data for tools abandoned by chimpanzees as well as counted the raw material available nearby. I conducted my research during five periods of fieldwork, totalling 140 days from 2014 to 2017 (12 Oct-12 Nov 2014; 16 Apr-12 May 2015; 14 Nov-14 Dec 2015; 13 Oct-12 Nov 2016; 08 May-31 May 2017). I collected all data, except for 26 days (16 Apr-12 May 2015) when data were collected by Katarina Almeida-Warren. Local field assistants collected data from 30 Nov 2016 to 17 Feb 2017.

The focus of my research was on 13 *Macrotermes* termite mounds. Mounds were randomly selected where chimpanzees have previously seen to fish for termites: 7 at Kasekela, 6 at Mitumba. Each study mound was given a unique identifier (GTMXXX for Kasekela, MITXXX for Mitumba) and was visited by the researcher every 1–3 days to monitor artefact presence. To avoid recording the same tool twice during repeated visits, artefacts were either collected and taken back to camp for closer scrutiny, or marked with a paint marker and left at the mound for taphonomical studies after measurement (Pascual-Garrido, in prep). At each mound, the maximum extension on either a north-south or east-west was measured with a meter tape (in cm) and a sample of at least 10 individual termite soldiers was collected and stored in a 1.5 ml vial filled with 85% ethanol. All samples were later identified by Rudolf H. Scheffrahn, University of Florida, USA. For all termite fishing tools, I recorded the following variables: 1. *Length*: measured with a meter tape (in cm); 2. *Width at midpoint*: measured with an electronic digital caliper (in mm); 3. *Tool material*: categories included *bark* (the outermost layer of tissue overlaying the wood of trees, shrubs, and vines that can easily peel lengthways in strips), *twig* (thin branches or stems of woody or non-woody herbaceous plants), *vine* (thin stems of non-woody herbaceous climber vines), or *grass* (the hollow vertical structural stems of grasses that provide support for flowers at the top and leaves attached at the nodes). Categories were chosen based on Pascual-Garrido^41^; 4. *Tool plant species*: source species were identified from the diagnostic characteristics of the implements, including type, sturdiness, color, and texture of tool material and associated leaves^41^. Source plants from which tools were manufactured were identified through the presence of scars left as result of chimpanzees removing raw material^41^. Samples from the source plants were curated in camp with a plant drier for later identification by Frank Mbago, Botany Department, University of Dar es Salaam, Tanzania.

At each study mound, I recorded the raw material availability (i.e. living plants). Using cardinal orientations (N-S, E-W), I divided the mound vicinity into four quadrants and arbitrarily selected the northwest 90°quadrant, a 5m circle around the mound for scrutiny^15,40,52^. For each surveyed quadrant, I counted the number and species of plants suitable as sources of raw material and the type of raw material that each could provide (bark, twig, vine, grass)^41^. Possible raw material types were chosen based on previous research of termite fishing tools^34,37^. Suitable raw materials were defined as long, thin, flexible pieces that the researcher could easily detach (twigs, vines, or grass) or peel off (bark) from the source plant using the hands and from which a suitable termite fishing tool could be made^40^.

No data were collected from chimpanzee individuals during this study. I recorded all chimpanzee artefacts, tool plant sources and termite mounds following site abandonment, with no direct physical contact with any of the chimpanzees. Research was conducted in accordance with relevant guidelines and regulations under the permits of Tanzania National Parks (TANAPA), Tanzania Wildlife Research Institute (TAWIRI), and Tanzanian Commission for Science and Technology (COSTECH). Research protocols were approved by Tanzania National Parks (TANAPA), Tanzania Wildlife Research Institute (TAWIRI), and Tanzanian Commission for Science and Technology (COSTECH).

### Statistical analyses

To test whether there were differences between communities in the number of tools recovered at the different mounds, I used a Generalized Linear Mixed Model with Poisson error function to control for the count nature of the dependent variable, using the ‘lme4’ package in R statistical software^75^. The model tested the number of tools as dependent variable, with the community and the mound size (defined as the maximum extension of the mound on either a north-south or east-west axis, z-standardised) as fixed effects, and the identity of each mound as a random effect to control for non-independence of data collected from the same mound over time.

To test whether tool characteristics (width, length) differed between communities while accounting for tool material, tool species, and mound properties, I fitted two Generalized Linear Mixed Models (one for each width and length) with Gaussian error function. The models included community, tool material, tool species, and mound size (z-standardised) as fixed effects, and the mound identity and the visit identity as random effects, to account for the fact that tools collected during the same visit and from the same mound are not independent. I restricted the tool species to *M*. *poggei* (166 cases), *D*. *lucida* (256 cases), and *G*. *forbesii* (49 cases), as only these species were used sufficiently by both communities, and restricted the tool material to bark (158 cases) and twigs (313 cases), for the same reason.

To test whether community had a significant effect in each model, I conducted full null model comparisons^76^ using a likelihood ratio test, where the null models included all variables except community. I tested the significance of effects by systematically dropping them from the models one at a time and comparing the resulting models with the full models using the “drop1” function in R. Multicollinearity between predictor variables (established using the Variance Inflation Factor implemented with the R package ‘car’^77^ was not an issue (maximum VIF = 2.4)^78^.

## Supplementary information

Dataset 1

## Data Availability

The datasets generated during and/or analysed during the current study are available from the author upon reasonable request.
