# Supplementary material for: Cultural variation between neighbouring communities of chimpanzees at Gombe, Tanzania
Source: Sci Rep. 2019 Jun 4;9:8260. doi: 10.1038/s41598-019-44703-4 (PMC6547654; doi:10.1038/s41598-019-44703-4)
Supplement: Supplementary file 1 — Dataset 1 [file 41598_2019_44703_MOESM1_ESM.pdf]

Cultural variation between neighbouring communities of chimpanzees at Gombe, Tanzania

Alejandra Pascual-Garrido[1]

[1]Primate Models for Behavioural Evolution Lab, Institute of Cognitive and Evolutionary Anthropology, School of Anthropology and Museum Ethnography, University of Oxford, 64 Banbury Road, Oxford OX2 6 PN, United Kingdom. Email: alejandra.pascual-garrido@anthro.ox.ac.uk

Supplementary Information

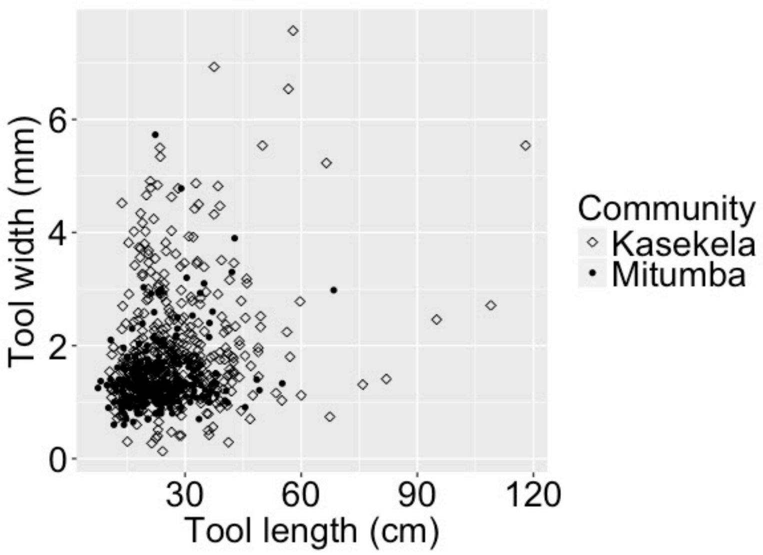

Supplementary Figure S1. Termite fishing tool dimensions in the two neighbouring communities at Gombe. Tool length and tool width for Kasekela community (white diamonds) and Mitumba community (black dots).

Supplementary Table S1. Termite fishing tool characteristics across tool species collected at Kasekela community and Mitumba community at Gombe.

Characteristics of Kasekela tools	Length (cm)				Width (mm)			
Tool Species	N	Mean	SD	Range	N	Mean	SD	Range
M. poggei	104	25.9	8.5	11-49.5	104	2.0	0.9	0.3-4.9
D. lucida	78	24.1	8.0	11.5-53.5	78	1.4	0.4	0.4-2.1
G. forbesii	36	40.8	23.3	14.7-118	36	3.4	1.4	1.0-6.9

Characteristics of Mitumba tools	Length (cm)				Width (mm)			
Tool Species	N	Mean	SD	Range	N	Mean	SD	Range
M. poggei	61	22.6	6.5	7.5-37.4	61	1.7	0.7	0.6-4.8
D. lucida	182	23.3	7.9	8.2-55.1	182	1.2	0.3	0.6-2.1
G. forbesii	13	32.5	14.0	15.8-68.4	13	2.8	1.2	1.0-5.7

Supplementary Table S2. Termite fishing tool characteristics across tool material types collected at Kasekela community and Mitumba community at Gombe.

Characteristics of Kasekela Tools	Length (cm)				Width (mm)			
Type of Material	N	Mean	SD	Range	N	Mean	SD	Range
Bark	195	29.4	12.9	11-109	194	2.5	1.3	0.3-7.6
Twig	179	26.5	12.7	10.5-118	179	1.4	0.6	0.1-5.5
Vine	25	29.3	12.2	11.8-57.1	25	1.6	0.5	0.8-3.5
Grass	5	34.4	11.8	16.8-47.5	5	1.6	0.5	1.1-2.4

Characteristics of Mitumba Tools	Length (cm)				Width (mm)			
Type of Material	N	Mean	SD	Range	N	Mean	SD	Range
Bark	45	25.0	10.0	10.7-68.4	45	2.3	0.9	0.9-5.7
Twig	222	23.1	7.8	7.5-55.1	222	1.2	0.3	0.6-2.6
